# Leveraging non-specialty personnel for mental health support in large-scale combat operations: A scoping review

**DOI:** 10.1080/08995605.2026.2620973

**Published:** 2026-02-06

**Authors:** Felicia N. Katzovitz, Julia G. Catanese

**Affiliations:** Daniel K. Inouye Graduate School of Nursing, Uniformed Services University of the Health Sciences, Bethesda, Maryland, USA

**Keywords:** Large-scale combat operations, austere environments, non-specialty personnel, psychological support, peer support

## Abstract

The shift from counterinsurgency to large-scale combat operations (LSCO) presents new challenges for delivering military mental healthcare, including resource limitations, disruptions to medical evacuation, and degraded network connectivity. The anticipated constraints in LSCO may delay access to specialty care, necessitating scalable approaches to address mental health care needs. To explore how mental healthcare needs have been addressed in similar contexts, this scoping review aims to chart the literature on interventions administered by personnel without formal mental health specialization or credentials in military or comparable operational settings. The review followed Arksey and O’Malley’s five-stage methodological framework to chart, collate, summarize, and report relevant findings. A systematic search strategy was used to identify literature from four databases (PubMed, CINAHL, PsycINFO, and Embase), and the authors conducted a full-text screening. Twenty-three studies met the inclusion criteria and informed the development of the conceptual model. Interventions were categorized by personnel role and mapped onto the Stress Continuum Model. The model offers a scalable, mission-adaptive approach that accounts for personnel roles and practice scopes. Rather than prescribing a fixed solution, the conceptual model provides leaders and medical planners with flexible, evidence-informed options. Findings illustrate how leaders, informal peers, trained peers, and non-specialty medical personnel may be incorporated into a role-based psychological support strategy for austere environments, including during LSCO.

**What is the public significance of this article?—**When specialty mental health providers are unavailable during large-scale combat operations (LSCO), non-specialty personnel, such as leadership, peers, and medics, can provide interventions to support their fellow service members. This scoping review organizes findings to help military leaders identify scalable mental health interventions that can be implemented using existing personnel and resources.

## Introduction

The transition from Middle Eastern counterinsurgency to large-scale combat operations (LSCO) represents a paradigm shift in U.S. mental health care delivery. During counterinsurgency operations, medical teams often operated from posts and bases supported by relatively secure supply lines, stable communication networks, and consistent medical evacuations (Dilday et al., 2024; Thomas, 2022). The steady operational tempo and efficient medical system were key contributors to historically low case fatality rates, with superior survival outcomes compared to earlier U.S. military conflicts (Howard et al., 2019; Remondelli et al., 2024). Medical planners structured medical and mental healthcare delivery to enable early interventions, efficient specialist access, and timely evacuations (Remondelli et al., 2024).

In contrast, analysts forecast LSCO environments marked by contested terrain, limited resources, unstable communication, prolonged patient holding, and casualty surges that may overwhelm current medical systems (Dilday et al., 2024; Thomas, 2022). While these conditions could strain all domains of medical care, mental health care is particularly vulnerable due to its reliance on early detection and rapid response (Paxton Willing et al., 2024).

### Mental healthcare in deployments

During counterinsurgency operations, mental healthcare delivery benefited from early detection, intervention, and medical evacuation following the introduction of embedded mental health providers (Deveney, 2024). Embedded mental health, also known as “embedded behavioral health,” refers to a care model within the U.S. military where mental or behavioral health providers are assigned, attached, or embedded within the organizational structure of operational military units, rather than within the military treatment facility (Deveney, 2024; Thrasher et al., 2024). The goal of embedding providers within units is to improve health outcomes through proximity, facilitated by shared unit culture and work schedules (Thrasher et al., 2024). While all U.S. military branches (i.e., the U.S. Army, Navy and Marine Corps, and Air Force and Space Force) use the embedded mental health care model, implementation varies across branches.

Mental or behavioral health providers may be assigned to U.S. Army brigades (Thrasher et al., 2024), U.S. Naval or Marine Corps divisions, ships, submarines, or groups (Hamlett & Reed, 2024), and operational U.S. Air Force line units using the Integrated Operational Support (Aycock et al., 2024). Additionally, the U.S. military uses Combat and Operational Stress Control (COSC) teams, which have been shown to promote return to duty and reduce stress (Maglione et al., 2022a). Interventions like embedded mental health services and COSC are supported by military leadership (Deveney, 2024), promote access to care, reduce stigma (Thrasher et al., 2024), and improve outcomes (Maglione et al., 2022a). However, anticipated LSCO constraints may complicate the delivery of these interventions.

The impact of disrupted embedded mental healthcare interventions was noted during the COVID-19 pandemic, as providers reported challenges with maintaining engagement during geographical separation. Technology-based interventions, such as telehealth and encrypted messaging platforms, were used to communicate with mental health care providers (Galloway & Martinez, 2025). Although virtual healthcare offers a confidential modality for delivering mental health care, challenges such as inconsistent policy oversight, reduced ability of providers to assess nonverbal cues, and technological limitations may negatively affect the quality and effectiveness of care (Galloway & Martinez, 2025). While these limitations were observed during the COVID-19 pandemic in comparatively stable environments, conditions in LSCO are expected to exacerbate these challenges.

### Potential impacts of LSCO conditions on mental healthcare delivery

Global threats, contested air and battlespace, and modernized weapon systems pose risks to the infrastructure required for telehealth delivery (Hoyt, 2025; Thomas, 2022). As a result, relying on technology-based mental healthcare solutions may not be an effective primary strategy. Disrupted network connectivity could further compound challenges in delivering mental healthcare in LSCO. The anticipated constraints, including degraded communication, prolonged evacuation timelines, and limited specialist availability, may significantly delay access to traditional mental health care services.

A recent Military Health System analysis proposed adaptations for future operations to address these anticipated constraints. The RAND Corporation recommended realigning medical forces to prepare for prolonged evacuations and to maximize treatment at the point of injury (Thomas, 2022). Hoyt (2025) argues that psychological support may not be delivered at the lowest possible level, reinforcing the need to expand training to incorporate updated models for an all-domain conflict. Given the operational demands and expected shortages of mental health specialists, identifying interventions and equipping non-specialty personnel to initiate and sustain psychological support in austere environments is crucial.

### Scoping review purpose

In response to an evolving operational landscape and recommendations to provide care closer to the point of injury, this review aimed to chart, collate, summarize, and report the literature on interventions administered by personnel without formal mental health specialization or credentials in military or comparable operational settings. The objective was to identify and characterize scalable interventions that could support psychological health when access to specialty care or telehealth is limited.

Guided by the PRISMA for Scoping Reviews (PRISMA-ScR) extension (Tricco et al., 2018), this scoping review used a systematic approach to include a wide range of literature and map evidence for application in an emerging field (i.e., mental healthcare delivery in LSCO environments) and to prepare for predicted operational changes (Peters et al., 2015). The review followed the five stages of the Arksey and O’Malley (2005) methodological framework, providing a structured method to chart, document, and map findings, promoting rigor and transparency. The five stages of the methodological framework are: Stage 1: Identifying the research question; Stage 2: Identifying relevant studies; Stage 3: Study selection; Stage 4: Charting the data; and Stage 5: Collating, summarizing, and reporting the results (Arksey & O’Malley, 2005).

#### Stage one: Identify the review question

The question guiding this review was: *How have military and civilian personnel without mental health specialization or credentials facilitated interventions that promoted psychological support, offering insights for addressing the unique challenges of LSCO?* The key concepts – population, intervention, and outcome – were derived from the traditional PICO format and were defined as (P)opulation: non-specialty military and civilian, (I)ntervention: feasible interventions for LSCO, and (O)utcome: psychological support. No (C)omparison element was specified since the aim was to identify and chart interventions rather than evaluate comparative effectiveness.

## Method

### Eligibility criteria

The eligibility criteria were established a priori, guided by Peters et al. (2015), Arksey and O’Malley (2005), and the PRISMA-ScR extension (Tricco et al., 2018). Studies were included if they involved a population that was not specialized, credentialed, licensed, or board-certified in mental health career fields. Interventions were included if they could be feasibly implemented during LSCO and yielded outcomes that promoted psychological support. Descriptive articles were included if the intervention met these criteria.

Studies involving populations not generalizable to LSCO (e.g., individuals who are incarcerated, residing in long-term care facilities, or are admitted to inpatient units for psychiatric conditions) were excluded. Interventions relying on specialty care in military treatment facilities, evacuation systems, or telemedicine were also excluded because these systems are likely to degrade during LSCO. Outcomes disconnected from the acute operational environment (e.g., purely longitudinal findings) and studies limited to prevalence reporting or gray literature (e.g., opinion pieces) were excluded.

### Stage two: Identify relevant studies

The search, conducted on 10 and 14 February 2025, was developed in collaboration with a medical librarian, was limited to English-language studies, and yielded the results presented in Table 1.

**Table 1.** Search strategies.

Database	Keywords and Subject Headings	Initial Results
PsycINFO	(‘non specialty mental health’ OR ‘peer support’ OR ‘peer intervention*’ OR ‘battle budd*’ OR ‘psychological first-aid’ OR wingman OR ‘general health care provider*’) AND (‘military health service’ OR ‘military health’ OR (military OR disaster*)) *Limiters*: Journal Articles, English Language, Adulthood	2/10/2025- 1928 results
Embase	(non-specialty-mental-health OR peer-support OR peer-intervention* OR battle-budd* OR psychological-first-aid OR wingman OR general-health-care-provider*):ab,ti,kw AND (‘mental health’/de/mj OR (mental-health OR psychological-support):ab,ti,kw) AND (‘military health service’/exp OR ‘military health’/exp OR (military OR disaster*):ab,ti,kw) *Limiters*: English	2/14/2025- 202 results
PubMed	(non-specialty-mental-health[tiab] OR peer-support[tiab] OR peer-intervention*[tiab] OR battle-budd*[tiab] OR psychological-first-aid[tiab] OR wingman[tiab] OR general-health-care-provider*[tiab]) AND (‘Mental Health’[mh] OR ‘Mental Health Services’[mh] OR mental-health[tiab] OR psychological-support[tiab]) AND (‘Psychology, Military’[Mesh] OR ‘Military Psychiatry’[Mesh] OR ‘Military Health Services’[Mesh] OR ‘Military Health’[Mesh] OR military[tiab] OR disaster*[tiab]) AND (english[Filter])	2/14/2025- 190 results
CINAHL	(‘non specialty mental health’ OR ‘peer support’ OR ‘peer intervention*’ OR ‘battle budd*’ OR ‘psychological first-aid’ OR wingman OR ‘general health care provider*’) AND MM(mental health or psychosocial support or emotional support saba ccc) AND (‘military health service’ OR ‘military health’ OR (military OR disaster*)) *Limiters*: English	2/14/2025- 21 results

*Note*. This table provides each database’s search strategies, keywords, and subject headings.

### Stage three: Study selection

All citations were uploaded into Covidence, an online review management platform that supports a range of evidence reviews by providing tools for citation import, duplicate removal, masked screening, full-text review, and conflict resolution (Covidence, 2025). Using Covidence ensured a consistent, auditable workflow among reviewers, thereby strengthening the rigor of the study selection process. The first author screened titles and abstracts using the predefined inclusion and exclusion criteria. Articles that met criteria underwent independent full-text screening and review by both authors, with discrepancies resolved through discussion. The selection process (see Figure 1) adhered to PRISMA-ScR guidelines (Tricco et al., 2018).

**Figure 1.**
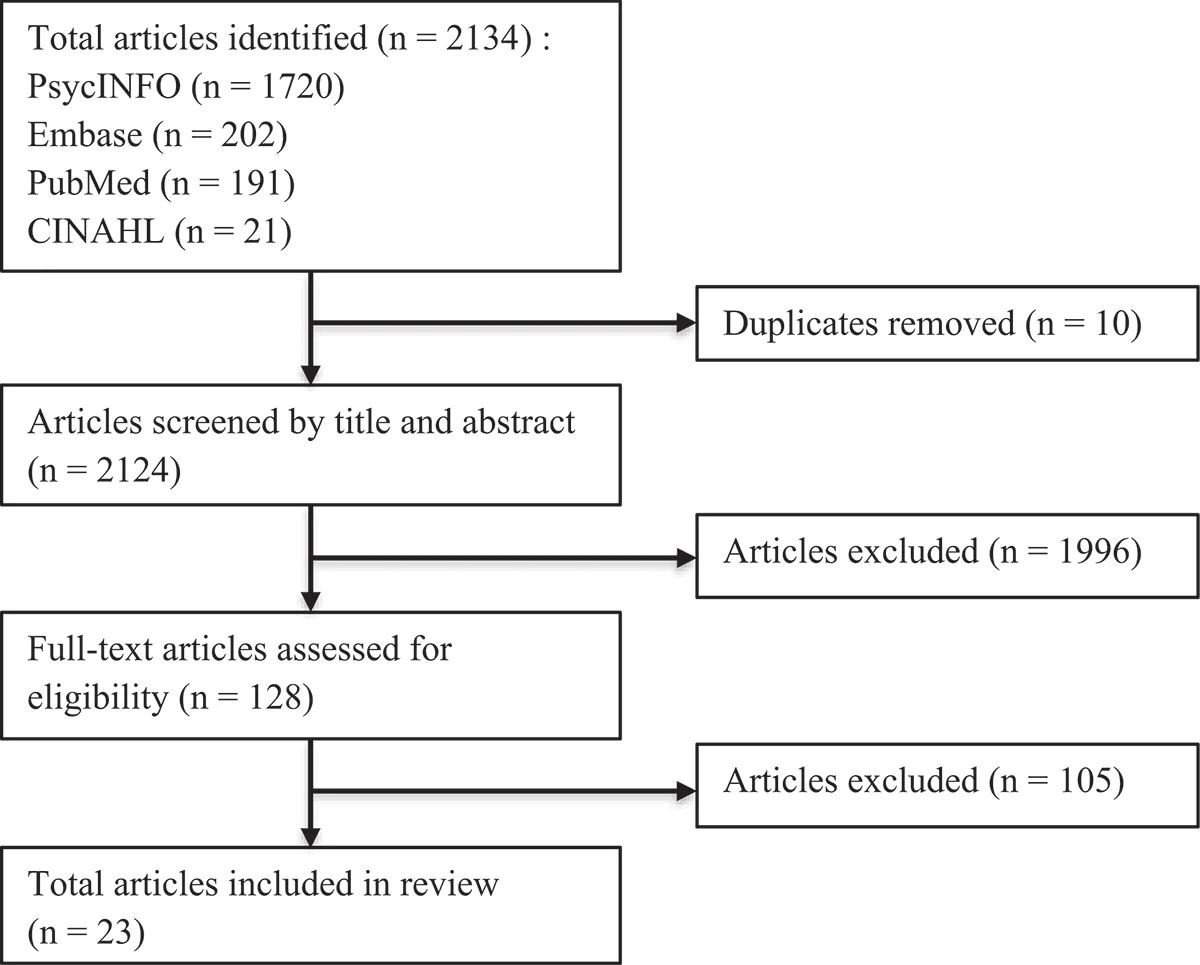
Search, screening, and selection strategy flow PRISMA diagram.

### Stage four: Charting the data

Following the full-text review, the authors conducted descriptive charting of study characteristics (*n* = 23) using a standardized form based on Arksey and O’Malley (2005). The charting process was essential for identifying patterns, gaps, and emerging themes, and for informing the development of the narrative synthesis and conceptual model. The following items were charted for each study: author(s), publication year and country, title, purpose or aims, intervention type and duration, methodology, sample size and characteristics, environment, mental health focus, comparator, outcome, findings, gaps or limitations, themes, and clinical or research recommendations. Images or graphics were also charted to inform the development of the conceptual model. This iterative process formed the foundation for data categorization and conceptual model development. See Table 2 for data charted on the first author, publication year, and country, intervention(s), and outcome(s) measure(s).

**Table 2.** Study characteristics.

First Author, Year, Country	Sample and Setting	Intervention(s)	Outcome(s)
**Leadership Tier**			
Albott, 2020, U.S.	Frontline workers in the COVID-19 pandemic (leadership-led program)	Battle Buddies	PTSD, burnout, stress exposure syndromes
Owen, 2020, U.S.	Leaders supporting nursing staff during post-crisis recovery	PFA and COSC	Resilience, emotional recovery, burnout prevention
Larsson, 2000, Sweden	NATO Implementation Force (IFOR) deployed to Bosnia	Post-trauma support (leader-led defusing sessions)	Post-deployment mental health status
**Informal Peer Tier**			
Wyman, 2022, U.S.	Air Force trainees at technical training bases	Wingman-Connect	Social integration, suicide prevention
Albott, 2020, U.S.	Frontline workers in the COVID-19 pandemic (peer-to-peer system)	Battle Buddies	PTSD, burnout, stress exposure syndromes
Villaruz Fisak, 2020, U.S.	Navy healthcare workers stationed overseas	Buddy Care	Occupational and caregiver stress, burnout, compassion fatigue, resilience
Greden, 2010, U.S.	Michigan Army National Guard post-deployment	Buddy-to-Buddy	Stigma, mental health treatment entry and adherence, suicidality
Jones, 2009, U.S.	Civilian disaster workers providing relief	CISM	Crisis response and compassion fatigue
**Trained Peer Tier**			
Khedr, 2024, Syria	Earthquake survivors in Northern Syria.	RAPID PFA	Distress, resilience, quality and meaning of life
Nordstrand, 2024, Norway	Norwegian Armed Forces deploying to Mali (UN mission MINUSMA) and conscripts with ~6 months of service	ReSTART	ASR
Knezevic, 2023, Australia	Healthcare staff after the Australian bushfires	Wellness Warriors	Perceived emotional support, recovery
Keyser, 2021, U.S.	Healthcare providers at a large hospital during the COVID-19 pandemic	PSP	Feasibility and acceptability, burnout, distress
Svetlitzky, 2020, Israel (Training evaluation)	Israeli combat soldiers in 12 combat battalions	YaHaLOM	Knowledge, confidence in managing ASR, stigma
Svetlitzky, 2020, Israel (Secondary analysis)	Israeli combat soldiers in 12 combat battalions (secondary data)	YaHaLOM	PTSD symptoms, stigma
Watson, 2018, U.K.	Civilian police forces	TRiM	Stigma, help-seeking, PTSD
Jones, 2017, U.K.	Military deployed to Afghanistan	TRiM	Help-seeking, mental health, perceived barriers
Smith-Osborne, 2017, U.S.	Army Reservists at battle assemblies	ASIST	Hopelessness, suicide risk factors, suicide ideation, suicide attempts, resilience
Everly, 2014, U.S.	Non-mental health personnel (including non-medical volunteers)	RAPID PFA	Knowledge to apply PFA, self-efficacy, resilience
Greenberg, 2010, U.K.	Sailors on twelve Royal Navy warships	TRiM	Psychological health, stigma, critical incidents
Schafer, 2010, U.S.	World Vision International staff in Haiti after an earthquake	PFA	Usefulness, application, limitations of PFA
**Medical Tier**			
Beatty, 2024, U.S.	Ukrainian military and civilian members who experienced wartime-related distress	ISP®	ASR, psychological distress, stabilization, resilience
Said, 2022, Palestine	Nurses working in trauma units in Palestine	RAPID PFA	Psychological preparedness, optimism, self-esteem, efficacy
Everly, 2014, U.S.	Non-mental health personnel (including healthcare professionals)	RAPID PFA	Knowledge to apply PFA, self-efficacy, resilience
Chandra, 2014, U.S.	Medical Reserve Corps volunteers	PFA (LPC)	Feasibility, confidence, perceived capability
Everly, 2010, U.S.	Nurses	PFA	Resilience, surge capacity

*Note*. COVID-19 = Coronavirus Disease 2019; PTSD = Post-Traumatic Stress Disorder; PFA = Psychological First Aid; COSC = Combat and Operational Stress Control; CISM = Critical Incident Stress Management; RAPID = Rapport, Assessment, Prioritization, Intervention, and Disposition; ReSTART = Registrer [register], Skap kontakt [establish contact], Tilby fellesskap [offer community], Avklar fakta [establish facts], Re-etabler tidslinje [reestablish timeline], and Tilbake til oppgave [return to task]; ASR = Acute Stress Reaction; YaHaLOM = Yetzirat kesher [connect], Hadgashat [emphasize], Levarer [inquire], Vidu [confirm], Matan [give]; TRiM = Trauma Risk Management; ASIST = Applied Suicide Intervention Skills Training; ISP® = Immediate Stabilization Procedure; LPC = Listen, Protect, Connect. Country designations reflect the country of origin of the study population rather than the country of journal publication. Some interventions (e.g., RAPID PFA) are listed across multiple tiers to reflect heterogeneous participant roles within the same study (e.g., non-medical volunteers and healthcare personnel), rather than duplicate study samples.

## Results

A total of 2134 articles were identified using a search strategy across four databases. After removing duplicates and applying eligibility criteria via title and abstract screening and full-text review, 23 studies were included in the descriptive analysis (see Figure 1).

### Stage five: Collating, summarizing, and reporting results

Following Arksey and O’Malley’s methodological framework, results were collated using a descriptive-analytic method rather than a formal synthesis. The aim was to provide a comprehensive overview of interventions, outcomes, and study characteristics, without synthesizing findings or appraising the quality of the evidence (Arksey & O’Malley, 2005). Findings are presented in Table 2 and are organized into four role-based tiers. The term “tiered” is used for organizational clarity, rather than to imply hierarchy or fixed boundaries.

### Study characteristics

The 23 included articles were published between 2000 and 2024. The studies were conducted with populations from the U.S. (*n* = 13), the U.K. (*n* = 3), Israel (*n* = 2), and one each in Syria, Norway, Australia, Palestine, and Sweden. Settings included military environments (Beatty et al., 2024; Greden et al., 2010; Greenberg et al., 2010; Jones et al., 2017; Keyser et al., 2021; Larsson et al., 2000; Smith-Osborne et al., 2017; Svetlitzky, Farchi, Yehuda, & Adler, 2020; Svetlitzky, Farchi, Yehuda, Start, et al., 2020; Villaruz Fisak et al., 2020; Wyman et al., 2022), disaster services (Jones & Majied, 2009; Khedr et al., 2024; Schafer et al., 2010), training exercises (Nordstrand et al., 2024), civilian medical organizations (Albott et al., 2020; Chandra et al., 2014; Everly et al., 2010; Knezevic et al., 2023; Owen & Schimmels, 2020; Said et al., 2022), civilian police forces (Watson & Andrews, 2018), and public health agencies (Everly et al., 2014).

### Analytic charting of interventions

The authors conducted exploratory charting and aggregated findings. As data were charted, distinct patterns emerged; differences in training level and influence formed the basis for a role-based conceptual model. The interventions were mapped on the Stress Continuum Model, based on the Combat Operational Stress First Aid (COSFA) manual’s Navy and Marine Corps Combat and Operational Stress Continuum model (Nash et al., 2010).

The Stress Continuum Model provides a military-informed framework for mitigating harm and promoting recovery, recognizing psychological health as a fluid process (Nash et al., 2010). Optimal functioning is defined as *Ready* (i.e., green), lower-risk mild distress is *Reacting* (i.e., yellow), higher-risk persistent distress is *Injured* (i.e., orange), and a clinical mental health disorder from unhealed stress is *Ill* (i.e., red) (Nash et al., 2010). The Stress Continuum Model was selected to contextualize each role-based intervention and align with psychological states.

#### Tiered model of intervention delivery roles

A conceptual model of delivery roles was developed by organizing interventions according to the operational scope of the personnel delivering them (see Table 2). The role-based model in this review aligns with recent LSCO guidance, which posits that effective psychological support in future, all-domain conflicts requires distributed capability across multiple levels, rather than relying solely on specialty providers (Hoyt, 2025). Commanders, leaders, or supervisors can deliver leadership-level interventions (see Figure 2).

**Figure 2.**
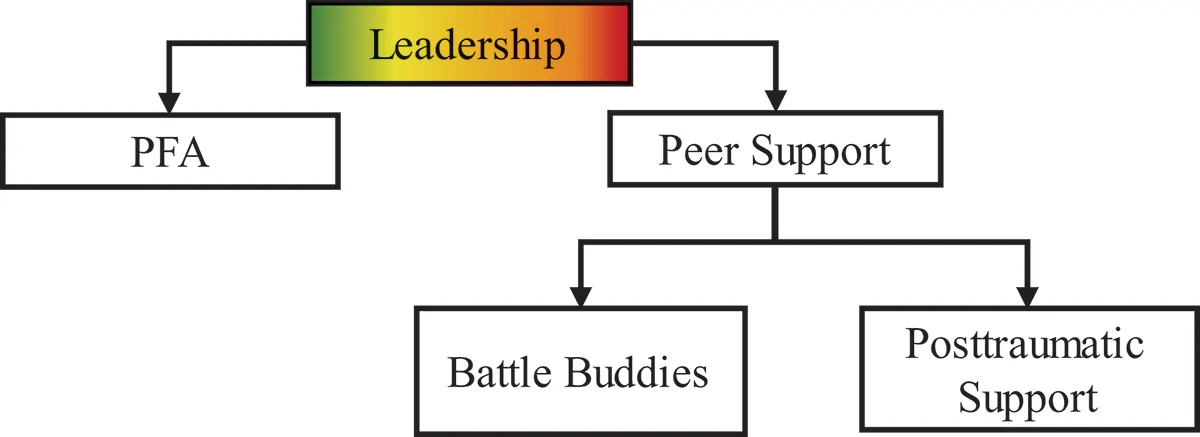
Leadership level interventions.

*Note*. PFA = Psychological First Aid.

##### Leadership

Leadership-level interventions were mapped across all stages of the Stress Continuum Model (i.e., *Ready* through *Ill)* to reflect leaders’ role in providing psychological support to service members experiencing stress at each stage (Nash et al., 2010). Examples include Psychological First Aid (PFA) (Owen & Schimmels, 2020), the Battle Buddy system (Albott et al., 2020), and leader-led defusing sessions as post-trauma support (Larsson et al., 2000). These interventions highlight the central role of supervisors and commanders in sustaining psychological support and resilience. These interventions enhance recovery after traumatic events by enabling leaders to provide structured, immediate, and visible support.

The PFA intervention equips leaders with eight evidence-informed core actions: contact and engagement; safety and comfort; stabilization; information gathering; practical assistance; connection with social supports; information on coping; and linkage with collaborative services, to create psychologically safe environments and promote help-seeking behaviors (Owen & Schimmels, 2020). The Battle Buddy system, adapted for healthcare workers during the COVID-19 pandemic, paired individuals with similar roles and stressors to foster trust, connection, and mutual validation, under leadership oversight (Albott et al., 2020). Similarly, Larsson et al. (2000) found that leader-led defusing sessions immediately following traumatic events more effectively supported long-term psychological outcomes than peer support alone. Leadership interventions leverage leaders’ authority, proximity, and visibility to model adaptive coping, reduce stigma, and facilitate early recovery, reinforcing unit cohesion and psychological health across military and civilian healthcare environments.

##### Informal peer

Informal peer-level interventions require minimal or no training (e.g., reading a document or hearing a briefing). Mapped to the *Ready* and *Reacting* zones of the Stress Continuum, these interventions enable peers to support one another before distressing symptoms escalate, reinforcing psychological support through trust and accessibility (see Figure 3).

**Figure 3.**
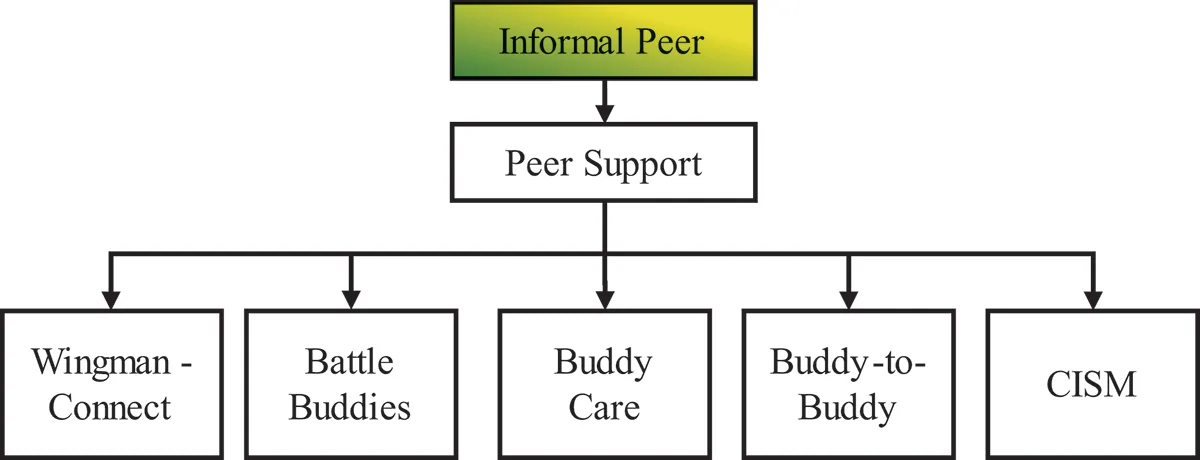
Informal peer level interventions.

*Note*. CISM = Critical Incident Stress Management.

Informal peer interventions of Buddy Care (Villaruz Fisak et al., 2020), Wingman-Connect (Wyman et al., 2022), Battle Buddies (Albott et al., 2020), Buddy-to-Buddy (Greden et al., 2010), Posttrauma Support (which includes peer support and defusing sessions), and Critical Incident Stress Management (CISM) programs (Larsson et al., 2000) emphasize spontaneous or lightly structured peer support systems embedded within military or high-stress occupational settings. These interventions provide preclinical support by fostering connection, encouraging emotional ventilation, and promoting psychological safety without formal training.

Villaruz Fisak et al. (2020) demonstrated that informal peer support reduced occupational stress in an overseas military population using a low-resource Buddy Care model. Similarly, Greden et al. (2010) showed that the Buddy-to-Buddy system increased mental health service engagement among veterans by pairing them with fellow service members to provide emotional and logistical support. Wyman et al. (2022) reported improved social integration and reduced suicidal ideation risk among airmen who participated in the Wingman-Connect program’s peer-driven contact strategy. Larsson et al. (2000) found that peer support following trauma, especially when combined with defusing sessions, improved mental health outcomes in deployed soldiers, without formal clinical intervention. While more structured, CISM includes informal peer defusing as a key component for mitigating compassion fatigue among disaster-exposed personnel (Jones & Majied, 2009). Together, these approaches demonstrate how timely peer interactions can help maintain well-being and encourage openness about mental health care.

##### Trained peer

Trained peer interventions are delivered by non-medical personnel who receive instruction before or during their deployments. Mapped to the *Reacting* zone of the Stress Continuum, these interventions (see Figure 4) extend behavioral health support by identifying and mitigating distress and referring individuals to medical personnel when needed.

**Figure 4.**
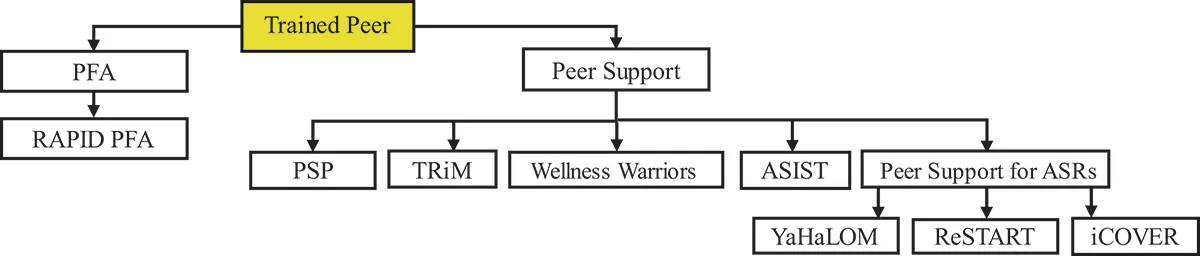
Trained peer level interventions.

*Note*. iCOVER = Identify, Connect, Offer commitment, Verify facts, Establish order of events, Request action; PFA = Psychological First Aid; RAPID = Rapport, Assessment, Prioritization, Intervention, Disposition; PSP = Peer Support Program; YaHaLOM = Yetzirat kesher [connect], Hadgashat [emphasize], Levarer [inquire], Vidu [confirm], Matan [give]; ReSTART = Registrer [register], Skap kontakt [establish contact], Tilby fellesskap [offer community], Avklar fakta [establish facts], Reetabler tidslinje [re-establish timeline], and Tilbake til oppgave [return to task]; TRiM = Trauma Risk Management; ASIST = Applied Suicide Intervention Skills Training.

The present review identified a rapid, peer-based intervention for acute stress reactions (ASRs) that has been adapted across multiple national military contexts. Initially developed by the Israel Defense Forces as YaHaLOM, the five-step protocol was designed to stabilize service members experiencing ASRs within high-stress environments (Svetlitzky, Farchi, Yehuda, Start, et al., 2020). The intervention was adapted to include a sixth step to support peers in identifying ASRs, while retaining the core framework, resulting in the U.S. adaptation, iCOVER (Adler et al., 2020, 2024), and the Norwegian Armed Forces’ ReSTART (Nordstrand et al., 2024). Across the adaptations, studies reported improvements in peer confidence, knowledge, and readiness to intervene when responding to ASRs (Adler & Gutierrez, 2022a, 2022b; Adler et al., 2020, 2024; Nordstrand et al., 2024; Svetlitzky, Farchi, Yehuda, Start, et al., 2020).

Other trained peer programs, such as Wellness Warriors (Knezevic et al., 2023), TRiM (Trauma Risk Management) (Greenberg et al., 2010; Jones et al., 2017; Watson & Andrews, 2018), PFA (Everly et al., 2010, 2014; Schafer et al., 2010), PSP (Peer Support Programs) (Keyser et al., 2021), and ASIST (Applied Suicide Intervention Skills Training) (Smith-Osborne et al., 2017), demonstrated similar outcomes. TRiM reduced stigma and promoted help-seeking (Greenberg et al., 2010; Jones et al., 2017; Watson & Andrews, 2018), while PFA (Everly et al., 2010, 2014; Schafer et al., 2010) and Wellness Warriors (Knezevic et al., 2023) showed that trained peer support can improve psychological safety and team resilience. Collectively, these interventions offer approaches for identifying distress and strengthening psychological support capacity within austere environments, such as during LSCO.

##### Medical

Medically trained personnel can deliver medical-level interventions (see Figure 5) without certification or specialization in mental health. Mapped primarily to the *Injured* and *Ill* zones of the Stress Continuum model, these interventions leverage medical personnel’s ability to integrate clinical judgment to offer rapid psychological stabilization.

**Figure 5.**
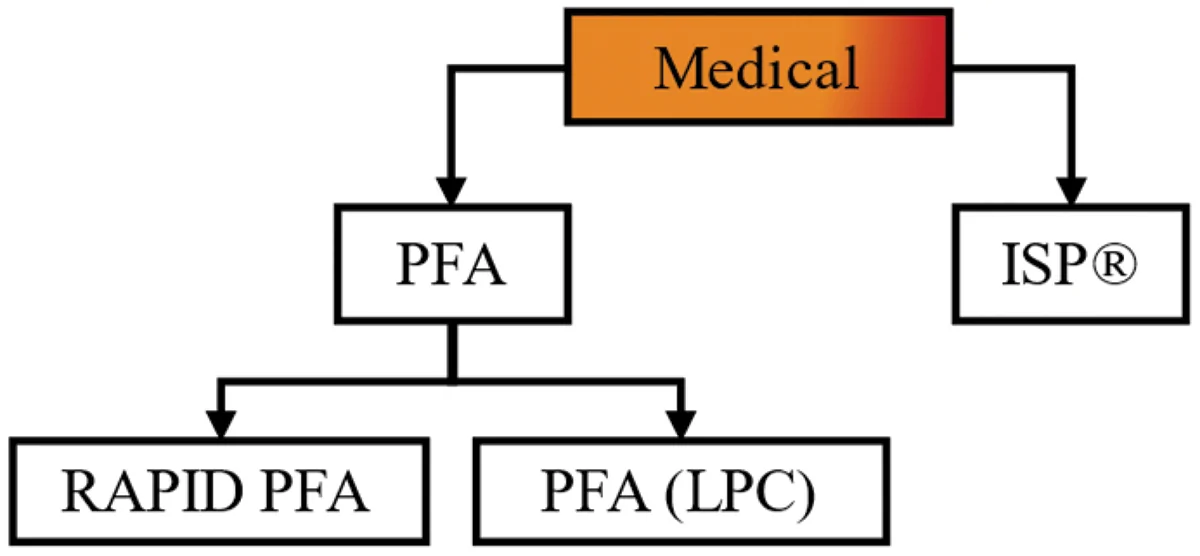
Medical level interventions.

*Note*. PFA = Psychological First Aid; RAPID = Rapport, Assessment, Prioritization, Intervention, Disposition; LPC = Listen, Protect, Connect; ISP® = Immediate Stabilization Procedure.

Medical personnel may employ distinct intervention approaches, including the Immediate Stabilization Procedure (ISP®) (Beatty et al., 2024) and PFA (Chandra et al., 2014; Everly et al., 2010; Said et al., 2022; Schafer et al., 2010), to support individuals experiencing psychological distress in high-stress or resource-constrained environments. The ISP® is an 8-phased, protocol-driven intervention designed for psychological stabilization in operational and high-threat environments (Beatty et al., 2024). The intervention requires minimal training and uses a scripted protocol with bilateral stimulation, defined as rhythmic, alternating left-right tactile leg or shoulder tapping delivered by the provider or self-administered by the service member (Beatty et al., 2024). Medical personnel can use ISP® for a range of responses, from mild distress to extreme overwhelm or “silent terror,” progressing through a series of paced, repetitive steps until the service member regains situational awareness and functional capacity (Beatty et al., 2024). The ISP®, developed for wartime and mass-casualty contexts, has demonstrated reductions in Subjective Units of Disturbance (SUD) levels and is feasible for delivery by medical personnel not specialized in mental health (Beatty et al., 2024).

Additionally, PFA has been integrated into disaster nursing (Everly et al., 2010), emergency humanitarian relief (Schafer et al., 2010), and public health volunteer training (Chandra et al., 2014; Said et al., 2022), consistently increasing provider confidence and intent to respond to distress. Although knowledge gains were modest (Chandra et al., 2014), these interventions improved psychological triage skills and enabled medical personnel to deliver empathic support and connect individuals to higher-level care when needed (Everly et al., 2010; Said et al., 2022; Schafer et al., 2010). Collectively, medical-level interventions expand mental health access and reinforce clinicians’ role in mitigating distress. Additionally, by using clinical judgment to determine when specialty mental health care is warranted, medical personnel help efficiently allocate limited mental health resources.

## Discussion

To the authors’ knowledge, this scoping review is the first to systematically examine, chart, and categorize mental health interventions delivered by non-specialty personnel in military or analogous contexts using a role-based model. This scoping review demonstrates how leaders, informal peers, trained peers, and non-specialty medical personnel can provide psychological support across the full spectrum of stress responses by mapping interventions onto the Stress Continuum Model (Nash et al., 2010). Findings informed the conceptual model (see Figure 6), which offers a scalable, mission-adaptive approach illustrating how each role supports individual psychological support across the stress continuum—*Ready*, *Reacting*, *Injured*, and *Ill*. While the leadership tier spans all phases as a continuous influence, the other three roles are primarily depicted within zones aligned with their training and scope. Although the roles are represented within zones, these boundaries are not exclusive; personnel may intervene as situational demands require, thereby bolstering mental health planning in austere environments.

**Figure 6.**
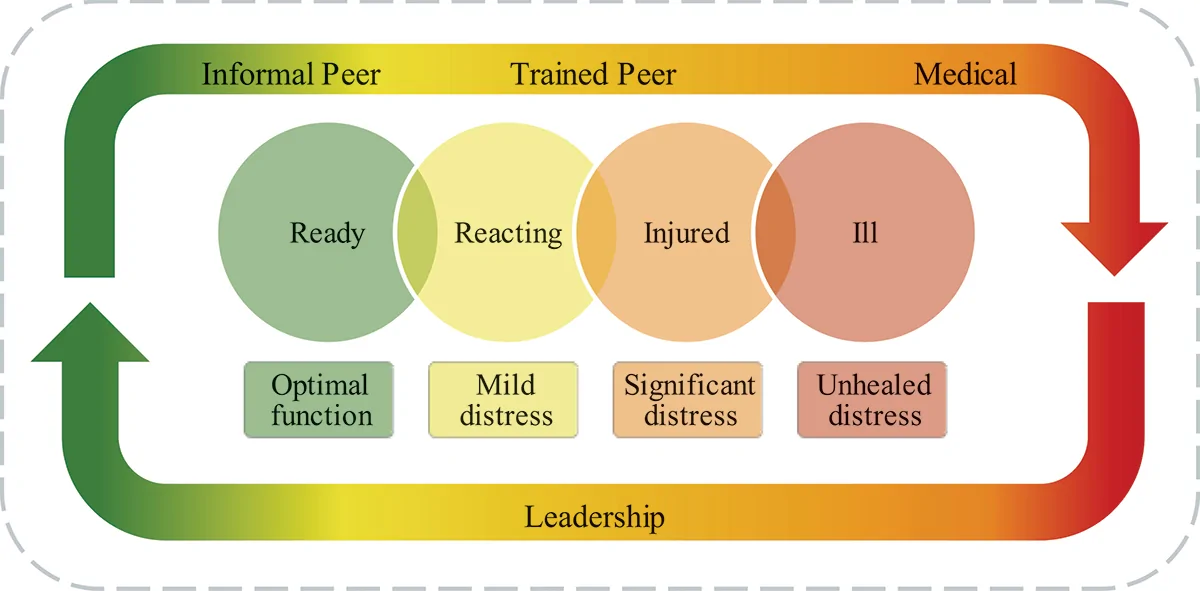
Dynamic non-specialty psychological support roles across the stress continuum.

*Note*. Consistent with the Stress Continuum Model (Nash et al., 2010), this conceptual model illustrates how personnel not specialized in mental health may provide psychological support in austere environments. Support roles are dynamic rather than prescriptive; placement reflects roles that may be well suited to specific stress states, while emphasizing that personnel may intervene across the continuum, depending on training, context, and operational need.

### Prior reviews of peer support interventions

Previous reviews have examined peer support interventions for personnel exposed to organizational stress (Fallon et al., 2023; Hooper et al., 2021; Maglione et al., 2022b). Fallon et al. (2023) reviewed peer support programs for public safety workers, identifying facilitators (e.g., confidentiality, trust, and shared experiences) and barriers (e.g., stigma) (Fallon et al., 2023). However, they did not examine interventions delivered by leadership or medical personnel. Hooper et al. (2021) reviewed individual-level interventions for healthcare workers, such as PFA and TRiM, whereas Maglione et al. (2022b) conducted a systematic review across military and first-responder populations; neither categorized interventions by role nor considered their applicability to austere environments.

Other articles have reviewed mental health interventions in disaster-related civilian settings (Castellano, 2021; Math et al., 2015; North & Pfefferbaum, 2013). North and Pfefferbaum (2013) emphasized the need for systematic triage to determine intervention needs but found inconclusive evidence for psychosocial interventions such as PFA and debriefing. However, the term “debriefing” warrants clarification as its meaning and implementation differ across contexts (Adler et al., 2009). In civilian literature, “psychological debriefing,” such as Critical Incident Stress Debriefing (CISD), typically consists of sessions held after a traumatic incident that include a mixture of psychoeducation, emotional ventilation, and trauma processing (North & Pfefferbaum, 2013). Military debriefings, such as Battlemind, integrate leadership, peers, and the individual’s strengths to reframe negative experiences, provide psychoeducation, and build on skills encouraged in military settings (e.g., relationship building) (Adler et al., 2009).

Recent studies (e.g., Hermosilla et al., 2023; Peng et al., 2024; Said et al., 2022) report support for psychosocial interventions. Math et al. (2015) advocated a preventive approach using cognitive-behavioral therapy and public health models. Castellano (2021) described the Reciprocal Peer Support Model in the context of large-scale disasters. While these reviews advance understanding of scalable interventions, they do not categorize interventions by roles.

Within the U.S. military-specific literature, Cooper et al. (2024) conducted a comprehensive scoping review of COSC programs, identifying 36 published evaluations of 19 programs across multiple U.S. service branches. The review applied a tiered, prevention-oriented framework to classify stress-control interventions while accounting for evidence levels (Cooper et al., 2024). However, interventions were not differentiated by provider role, nor was their feasibility examined in operationally constrained environments. More recently, Hoyt (2025) proposed a conceptual model emphasizing “point-of-injury” psychological support; although highly informative, the work did not systematically map specific interventions. The present review extends these efforts by systematically mapping interventions to provider roles and considering how these roles may function across the Stress Continuum in austere environments.

### Recommendations for practice

The synthesized findings provide key strategies to operationalize role-based psychological support in austere environments during LSCO. Implementing the role-based model in Figure 6 requires multi-level preparation, training, and commitment.

#### Leadership as a continuous factor

The model positions leadership across all phases, underscoring leaders’ roles in encouraging help-seeking, promoting early intervention, and fostering the command climate (Hoyt, 2025). Leaders are uniquely positioned to shape unit culture, as their behaviors can reinforce stigma and barriers to mental healthcare (Britt et al., 2012). Because of their proximity, leaders can apply principles of PFA and COSC to recognize stress early and adjust workflows that better meet service members’ needs (Larsson et al., 2000; Owen & Schimmels, 2020). In austere, psychologically demanding environments, leaders can make informed operational changes, such as adjusting sleep-work cycles, redistributing workload, and implementing unit-level interventions that may help preserve the force (Hoyt, 2025). Equipping leaders to provide psychological support effectively is critical; leader preparation could include brief training to model adaptive coping strategies, recognize ASRs, and intervene appropriately (Adler et al., 2024). Incorporating skill training into pre-deployment courses for leaders may strengthen leaders’ capacity to provide early, continuous psychological support in austere environments.

#### Empowering informal peer support

Peers without formal training can provide meaningful psychological support grounded in collective identity, unit cohesion, and proximity, as shared experiences may strengthen trust and promote conversations about mental health (Pfeiffer et al., 2012). Lightly structured dyad or peer-based models have demonstrated that pairing service members can strengthen psychological safety, promote early distress monitoring, reduce stigma, and assist with navigating barriers to mental healthcare, which is crucial in austere environments during LSCO (Albott et al., 2020; Greden et al., 2010; Villaruz Fisak et al., 2020; Wyman et al., 2022). In settings where communication may be degraded and specialty mental health care providers are unavailable, equipping peers with brief, practical tools (e.g., quick-reference guides, ASR identification cards, or conversation prompts) may build confidence in supporting teammates with early signs of distress and expand an organization’s capacity for psychological support.

#### Integrating role-based mental health planning into pre-deployment training

Medical planners can include mental health training in pre-deployment preparations. Incorporating trained-peer programs expands access to early mental health interventions, especially in austere environments. Units can integrate scenario-based rehearsals into pre-deployment training to familiarize service members with recognizing ASRs and responding appropriately, thereby expanding psychological support capacity (Hoyt, 2025). An opportunity for these preparations is to incorporate peer-based intervention protocols (i.e., iCOVER, YaHaLOM, and ReSTART) into the pre-deployment training (Adler & Gutierrez, 2022a, 2022b; Adler et al., 2020, 2024; Nordstrand et al., 2024; Svetlitzky, Farchi, Yehuda, Start, et al., 2020).

#### The role of medical personnel not specialized in mental health

Medical personnel are often first responders to physical and psychological concerns. Although not formally trained in mental health, their integrated role in healthcare allows them to recognize distress and provide early support. Brief training could expand access to care and support intentional, timely referral to specialty services when clinically indicated, thereby conserving limited resources. The findings from this review suggest that medical personnel can be equipped with protocol-driven tools, such as the ISP® (Beatty et al., 2024) or PFA (Chandra et al., 2014; Everly et al., 2010; Said et al., 2022; Schafer et al., 2010), to offer psychological support in resource-limited or high-threat environments. However, evidence for ISP® is currently limited to feasibility and short-term outcomes, underscoring the need for longitudinal evaluation. Training medical personnel, such as medics, corpsmen, and nurses, on psychological support interventions may bridge peer support and specialized mental health care. Embedding these skills into routine rehearsals and pre-deployment trainings may normalize psychological stabilization as part of standard care, ensuring continuity when specialists are unavailable.

### Recommendations for future research

Future research may build on the model developed in this review by conducting systematic investigations of intervention implementation with formal quality appraisal. Research is needed to evaluate which components of peer-based interventions for ASRs (i.e., iCOVER, YaHaLOM, and ReSTART) and medical interventions (i.e., ISP® and PFA) are effective in austere environments, given anticipated LSCO constraints. Additionally, future studies may evaluate how role-based mental health planning would be implemented into pre-deployment training.

### Limitations

Several limitations warrant mention. Consistent with the methodological framework detailed by Arksey and O’Malley (2005), the review did not include a formal quality appraisal, as the aim was to chart, collate, summarize, and report interventions rather than to evaluate methods. Consequently, the strength of the evidence was not formally assessed, and the findings should be interpreted as descriptive and exploratory. Additionally, because intervention families (e.g., iCOVER, YaHaLOM, and ReSTART) may be indexed under different terminology in the literature, some related programs (e.g., iCOVER) may not have been adequately captured during the initial database screening. This reflects a common challenge in scoping reviews, which map broad concepts across heterogeneous sources and terms. Furthermore, the review was restricted to four English-language databases and excluded gray literature, limiting its scope. Finally, recent literature recommends validating the Stress Continuum Model (Ram et al., 2024), which underpins this review’s conceptual model. While the role-based model provides structure, it should be validated in future studies. Programs that did not appear in the eligible literature, such as broad mental health literacy training programs like Mental Health First Aid, may be relevant but were not included because no published studies met the present review’s inclusion criteria.

## Conclusions

This review identified 23 studies describing interventions that non-specialty personnel could deliver and potentially implement during LSCO. The review mapped, collated, summarized, and reported the findings using Arksey and O’Malley’s (2005) methodological framework. Through charting, the authors developed a role-based conceptual model mapped onto the Stress Continuum Model (Nash et al., 2010) that illustrates how non-specialty personnel can provide psychological support across the stress spectrum. Findings suggest that leaders, informal peers, trained peers, and medical personnel can form an integrated psychological support system in austere environments during LSCO. The tiered model provides military leaders with a flexible model for adapting mental health support strategies to mission demands and for promoting broader psychological support.

## Data Availability

The data that support the findings of this study are available from the corresponding author, FNK, upon reasonable request.
